# Vertical and horizontal integration of multi-omics data with miodin

**DOI:** 10.1186/s12859-019-3224-4

**Published:** 2019-12-10

**Authors:** Benjamin Ulfenborg

**Affiliations:** School of Bioscience, University of Skövde, Skövde, Sweden

**Keywords:** Multi-omics, Data analysis, Data integration, Transparency

## Abstract

**Background:**

Studies on multiple modalities of omics data such as transcriptomics, genomics and proteomics are growing in popularity, since they allow us to investigate complex mechanisms across molecular layers. It is widely recognized that integrative omics analysis holds the promise to unlock novel and actionable biological insights into health and disease. Integration of multi-omics data remains challenging, however, and requires combination of several software tools and extensive technical expertise to account for the properties of heterogeneous data.

**Results:**

This paper presents the miodin R package, which provides a streamlined workflow-based syntax for multi-omics data analysis. The package allows users to perform analysis of omics data either across experiments on the same samples (vertical integration), or across studies on the same variables (horizontal integration). Workflows have been designed to promote transparent data analysis and reduce the technical expertise required to perform low-level data import and processing.

**Conclusions:**

The miodin package is implemented in R and is freely available for use and extension under the GPL-3 license. Package source, reference documentation and user manual are available at https://gitlab.com/algoromics/miodin.

## Background

With the advances in high-throughput biotechnology over the past two decades, we now have access to an unprecedented wealth of data for many omics modalities. In this era of biomedical big data, the primary research challenges are how to integrate and analyze large-scale data of different types and sources to gain new insights into the complex mechanisms behind health and disease [1–4]. In a study by Woo et al., DNA copy-number variation, methylation and gene expression were profiled in a cohort of hepatocellular carcinoma (HCC) patients. Integrative omics analysis revealed three molecular subtypes of HCC with differences in prognostic outcomes [5]. Zhu et al. performed a comprehensive pan-cancer integrative analysis showing that a combination of clinical variables with molecular profiles improved prognostic power in 7 of the 14 cancer types studied [6]. Lau et al. carried out a cardiac hypertrophy study in mice based on transcriptomics, proteomics and protein turnover data. The combination of multi-omics data revealed complementary insights into the pathogenesis of the disease [7]. These and other studies show that the integrative approach deliver novel biological insights. Advanced bioinformatics tools and algorithms have been developed that can analyze multiple modalities of omics data [8–10], but performing transparent and reproducible integrative analysis remains a significant challenge. Notably, considerable technical expertise is required to use many tools and combine them into a coherent pipeline.

Bioconductor is one of the largest open source projects for analysis of omics data [11], hosting more than 1600 software packages as of release 3.8. Many experimental techniques (e.g. microarrays, sequencing and mass spectrometry) and omics data types (e.g. genomics, transcriptomics and proteomics) are supported [12–20]. To perform data analysis, the project hosts many packages for different workflow steps, such has import, annotation, pre-processing, quality control, statistical analysis, biological interpretation and visualization [12, 21–26]. By promoting a common set of data structures, package interoperability, version control, extensive documentation and high development standards, the project contributes significantly to distributing R software in bioinformatics. Furthermore, Bioconductor hosts experimental data, workflows, tutorials and other materials to facilitate learning, usage and combination of packages. With its large and active community, Bioconductor continues to expand to meet the future challenges in multi-omics data analysis.

Given the functionality it provides, Bioconductor is an obvious choice when selecting software for performing integrative multi-omics data analysis. However, even for seasoned bioinformaticians a lot of technical expertise and work is required to combine packages into coherent pipelines. Knowing which packages to use is an issue when working with new techniques and data, since there are several possible packages available for a given problem. Learning how to use several packages takes time, given the need to be familiar with the logic behind data structures along with multiple functions and their parameters. Another challenge is the growth in complexity of the analysis scripts, where code is required to perform every analysis step, including import, pre-processing, quality control, statistical analysis and interpretation. This problem is exacerbated when working with multi-omics data and performing integrated analysis, where several steps are needed for every experimental technique and data type. This increases the risk of clerical errors and results in low transparency in terms of what processing and analysis steps that have been performed.

A related problem in omics data analysis is the lack of a systematic way to specify generic study designs in analysis scripts. Issues may include what experimental variables to analyze, how to define sample groups and statistical comparisons, how samples are paired, how to correct for batch effects and how to collapse replicates by mean. This can be performed ad hoc with e.g. variables and indexing operations, but this is error prone and gives low transparency when dealing with large datasets, multiple data types and more complex designs. Another general problem is the reproducibility of bioinformatics workflows [27, 28], i.e. to ensure that the same results are obtained when running a workflow on the same data with the same steps and parameters. This has been addressed by Nextflow [29] and related software, which are used to construct workflows and support Docker technology [30] for deployment. This technology ensures that the analysis environment remains the same and protects against numeric instability across different systems. The BiocImageBuilder [31] is a tool designed to promote reproducibility of Bioconductor workflows by building a Docker image configured with all necessary software. The image also supports JupyterHub [32] and Binder [33] for private and public deployment of Jupyter notebooks for sharing and rerunning the analysis.

Several tools and techniques have been developed for integrative analysis of multi-omics data [34, 35]. One popular approach is matrix factorization, where the objective is to infer latent factors that explain interpatient variance within and across omics modalities. Multi-Omics Factor Analysis (MOFA) is an unsupervised matrix factorization technique that is a generalization of Principle Component Analysis to several data matrices. Two strengths of MOFA are that it can integrate data from different distributions and handle missing data [36]. The mixOmics package [37] provides both unsupervised and supervised methods based on Partial Least Squares and Canonical Correlation Analysis, with generalizations to multi-block data. Another powerful approach to data integration is graph-based clustering of samples, which has been applied to disease subtype discovery. In Similarity Network Fusion, single-omics patient similarity networks are constructed, followed by iterative exchange of information to generate a fused multi-omics patient network [38]. Another technique relies on permutation-based clustering and was developed to identify robust patient partitions. This method integrates data by detecting the agreement between omics-specific patient connectivity [39].

To address the challenges of multi-omics data analysis, the miodin (MultI-Omics Data INtegration) R package was developed. The package provides a software infrastructure to build data analysis workflows that import, process and analyze multi-omics data. Workflows accommodate data from different omics modalities, including transcriptomics, genomics, epigenomics and proteomics, and from different experimental techniques (microarrays, sequencing and mass spectrometry). The package allows users to integrate omics data from different experiments on the same samples (vertical integration) or across studies on the same variables (horizontal integration). Furthermore, the user is provided with an expressive vocabulary for declaring the experimental study design, to render this explicit within the analysis script and reduce the risk of clerical errors. A key design goal when developing miodin was to streamline data analysis, by providing a clean syntax for building workflows and minimizing the extent of technical expertise required to combine multiple software packages. The motivation behind this was to promote transparent biomedical data science.

## Implementation

### Package overview

**Fig. 1:**
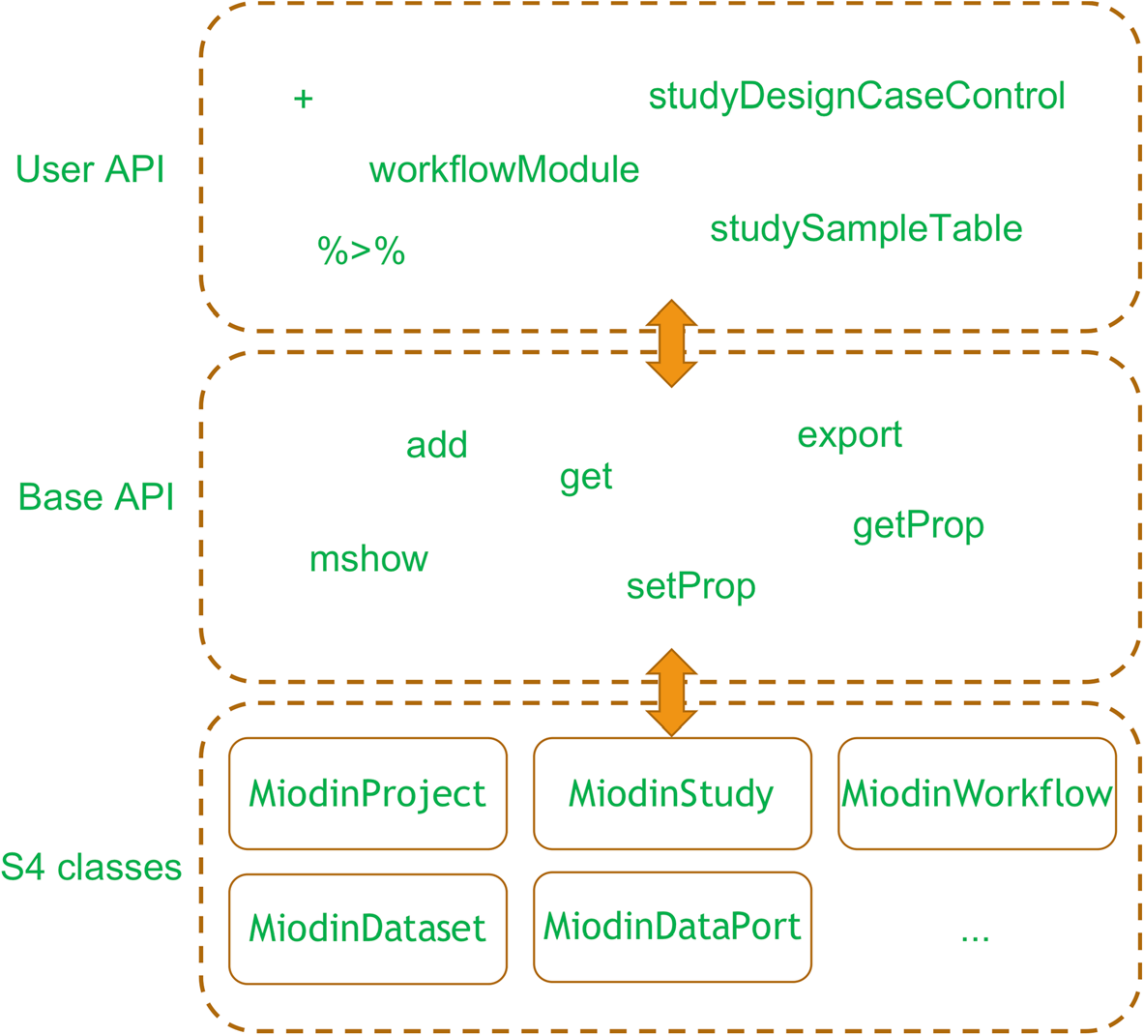
Package design. The miodin package provides a software infrastructure for data analysis implemented as a set of S4 classes. The base API contains standard generics for object manipulation and the user API provides convenience functions to facilitate package usage

The miodin package was implemented following the S4 object-oriented programming paradigm. Infrastructure functionality is supported by 16 S4 classes for which a common set of standard generics (base API) has been defined. Apart from the base classes, a number of workflow module classes have been developed, which serve as the building blocks of workflows. On top of the base API is a high-level user API consisting of convenience operators + and % > % along with helper functions to simply manipulation of objects (Fig. 1). The user API has been developed to reduce the learning curve for the package and minimize the number of classes, functions and parameters the user needs to learn.

**Fig. 2:**
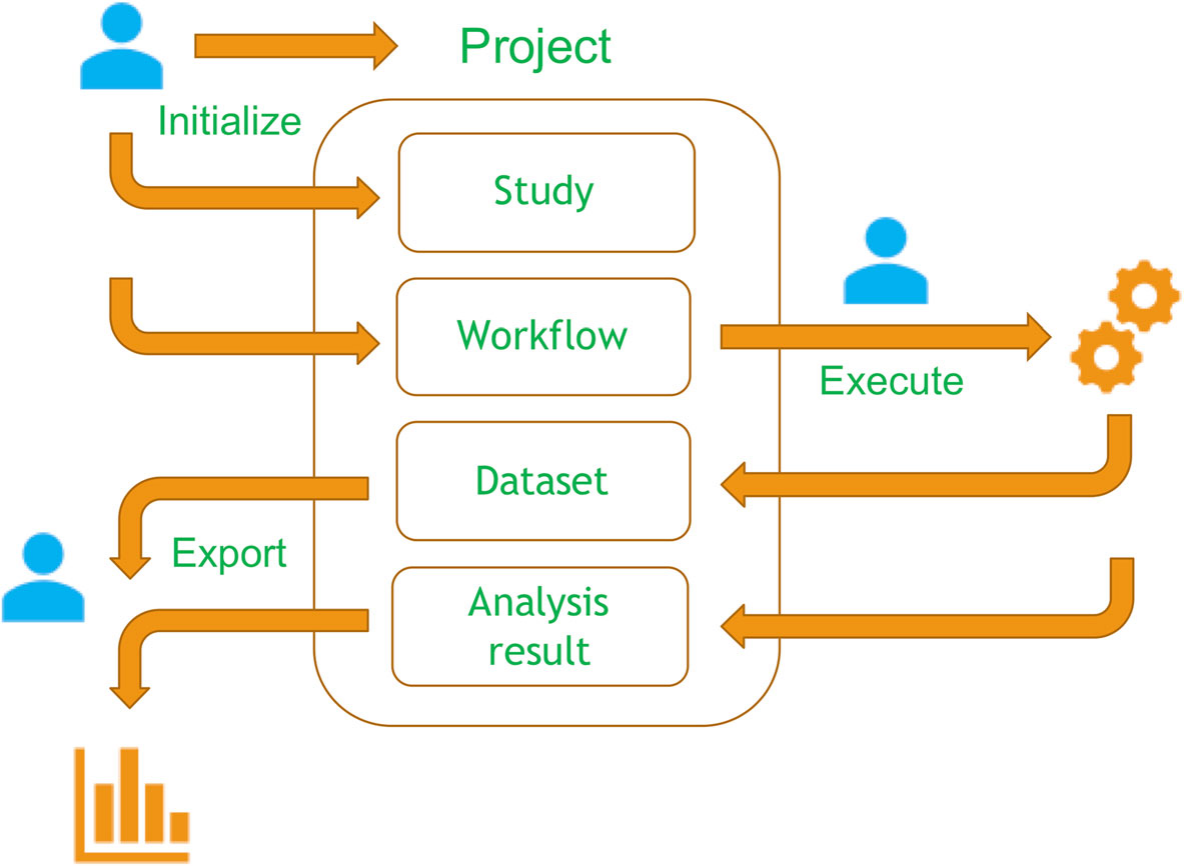
Data analysis in miodin. The user starts by defining a project, a study and a workflow. The study contains the design of the experiment and the workflow is defined by instantiating analysis modules, which generate datasets and analysis results upon execution. The user can then inspect and export the data and results

Data analysis with miodin follows an intuitive three-step process illustrated in Fig. 2. The user first initializes a project, a study and a workflow. The project serves as a placeholder for all other objects and the study is used to declare the study design, including what samples and assays to analyze, and the experimental variables of interest, if any. The miodin package implements an expressive study design vocabulary and several convenience functions for common designs, such as case-control and time series experiments. These allow the user to declare all information required for data analysis in one place, thus reducing the risk of clerical errors in the analysis script and the amount of information the user must provide during the analysis itself. The workflow is used to build the data analysis procedure as a set of sequentially connected workflow modules that carry out specific tasks, such as data import or processing. The analysis is performed by executing the workflow, which generates datasets and results. These can be inspected, exported and used for further analysis.

### Study design vocabulary

Information related to a study is managed using the MiodinStudy class. The study design can be declared manually by instantiating an empty study and using helper functions that add different properties to the design (Table 1) or using convenience functions available for some of the most common designs (Table 2).

**Table 1:** Study design helper functions

Function	Description
studySamplingPoints	Set the sampling points (e.g. time points)
studyFactor	Define a factor (experimental variable)
studyGroup	Define a sample group based on existing factors
studyContrast	Define a contrast (sample group comparison)
studySampleTable	Add a table with sample annotation data
studyAssayTable	Add a table with assay annotation data

**Table 2:** Common study design functions

Function	Description
studyDesignCaseControl	Single factor dividing samples into two groups
studyDesignMultipleGroups	Single factor dividing samples into multiple groups
studyDesignRepeatedMeasures	Single factor and multiple sampling points
studyDesignTwoFactors	Two factors and multiple sampling points

The purpose of declaring the study design is for the user to give an explicit definition on what samples are included (in a sample table), what assays or experimental data files to analyze (in an assay table), what sample groups exist and which groups to compare during the analysis. The benefits of this are that the user can provide all this information in one place in the analysis script and that no variable manipulation is needed on the user’s part. Furthermore, when the user adds sample and assay tables these are automatically validated against the declared study design to detect potential clerical errors that might otherwise disturb the downstream analysis. For detailed examples how to declare the study design, see the miodin user manual in the GitLab repository (https://gitlab.com/algoromics/miodin).

### Workflow syntax

When the study design has been declared the next step is to define the data analysis workflow. A workflow is built by instantiating the MiodinWorkflow class and adding workflow modules to it, each one performing a specific task. Workflow modules are added to the workflow object by + operator and a module-specific instantiation function. To feed the output from one module as input to the next, they can be combined using the pipe operator % > %.

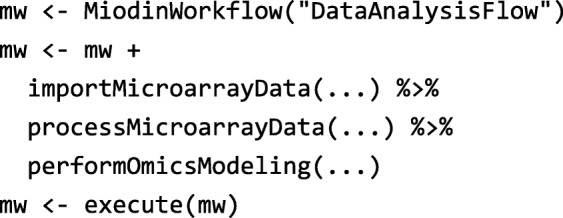



This script initializes a workflow called DataAnalysisFlow with three workflow modules. Module parameters have been omitted for brevity. The first module imports microarray data, the second processes the output from the first module, and the final module performs statistical testing on the processed data.

The analysis is carried out by calling the execute method. This syntax allows the user to define streamlined data analysis workflows, enhancing readability of the analysis script compared to longer chunks of code. Workflow modules have names starting with verbs denoting their function, making them easier to remember and improving analysis transparency. To further improve transparency, the analysis workflow automatically documents each processing and analysis step, including a description of what was done, what function was called, the name and version of the package, names and values of parameters, and how this affected the data. These can be inspected and exported as part of the dataset, thus addressing the issues of provenance [27], which is one important aspect of reproducibility.

### Package features

The workflow modules available as of miodin version 0.4.1 are described in Table 3. Import, processing and analysis of data is supported for different experimental techniques and omics data types as given in Table 4. For microarrays, raw data from Affymetrix arrays (CEL format) and Illumina arrays (IDAT format) is supported, including transcriptomics, genomics (SNP) and methylation data. Processed data is also supported for microarrays, sequencing (RNA-seq counts) and mass spectrometry (protein quantification). Workflow modules store data in instances of the MiodinDataset class, which inherits from MultiDataSet [40]. The MultiDataSet class provides functions to combine data from different omics-specific objects (e.g. ExpressionSet and SummarizedExperiment) and recover the original objects later on. MiodinDataset includes additional slots to hold processed data, interactions, quality control reports, processing protocols and auxiliary data. Table 5 lists the R package dependencies of miodin used for bioinformatics analysis.

**Table 3:** Workflow modules

Function	Description
downloadRepositoryData	Downloads data from an online repository
importMicroarrayData	Imports raw microarray data from Affymetrix and Illumina arrays
importProcessedData	Imports processed RNA, SNP, methylation and protein data
processMicroarrayData	Pre-processes microarray data
processSequencingData	Pre-processes sequencing data
processMassSpecData	Pre-processes mass spectrometry data
integrateAssays	Integrates several datasets into one
performFactorAnalysis	Performs factor analysis by matrix factorization
performHypothesisTest	Performs hypothesis testing
performLinearModeling	Performs generalized linear modeling with snpStats
performOmicsModeling	Performs modeling with limma, DMRcate or edgeR depending on the input object

**Table 4:** Supported experimental techniques and data types

	RNA	SNP	Methylation	Protein
Microarray	Raw and processed	Raw and processed	Raw and processed	
Sequencing	Processed			
Mass spectrometry				Processed

**Table 5:** Package dependencies

Package	Description
AffyCompatible	Annotation of Affymetrix microarrays
ArrayExpress	Access to the ArrayExpress online repository
crlmm	Genotyping of microarray SNP data
DESeq2	Processing of RNA-seq data
DMRcate	Statistical analysis of methylation data
edgeR	Statistical analysis of RNA-seq data
ff	Store large in-memory datasets on disk
limma	Statistical analysis of microarray RNA data
minfi	Import and normalization of microarray methylation data
mixOmics	Methods for integrative analysis of multi-omics data
MOFA	Integrative analysis by multi-omics factor analysis
MSnbase	Import of proteomics data
MultiDataSet	Data integration of multi-omics data
oligo	Import and normalization of microarray RNA data
RefFreeEWAS	Correction for cell type composition in methylation data
SNPRelate	Processing SNP data
snpStats	Statistical analysis of SNP data
SummarizedExperiment	Import of RNA-seq data
wateRmelon	Normalization of microarray methylation data

### Omics data integration

The miodin package can be used for analysis single-omics data, though by design the package is intended to streamline multi-omics data integration and analysis. Two case studies were carried out to illustrate how horizontal integration (across studies) and vertical integration (across omics modalities) can be performed. For demonstration purposes, data used in the case studies were pre-processed and included in the companion package miodindata prior to analysis with miodin. For horizontal integration, three lung cancer transcriptomics datasets with accession number E-GEOD-27262 [41], E-GEOD-19188 [42] and E-GEOD-40791 [43] were downloaded from ArrrayExpress [44]. Probes were mapped to genes with NetAffx file HG-U133-Plus-2-NA36 and each dataset was filtered to include only the first 2000 genes. Vertical integration was carried out using breast cancer data from the curatedTCGAData package [45]. RNA-seq gene and miRNA count data as well as 450 k methylation data were included in the analysis. RNA-seq gene and methylation data were filtered to include only the 5000 top-variance features.

## Results

### Horizontal integration: meta-analysis on lung cancer transcriptomics data

To perform meta-analysis in miodin, a study design must be declared for every dataset included in the analysis. This implies defining a sample table and assay table (as data frames) and calling the appropriate study design function. The three transcriptomics datasets used here (referred to as Wei2012, Hou2010 and Zhang2012) have case-control designs and were declared using studyDesignCaseControl (see Additional file 1). The Wei2012 dataset contained 50 samples; 25 from stage 1 lung adenocarcinoma tissue and 25 paired samples from adjacent normal tissue [41]. Sample pairedness was specified with the paired argument to the study design function, naming a column in the sample table containing information of how samples are paired. The Hou2010 dataset contained 156 samples (91 tumors and 65 healthy controls) and Zhang2012 contained 194 samples (94 tumors and 100 healthy controls).

**Fig. 3:**
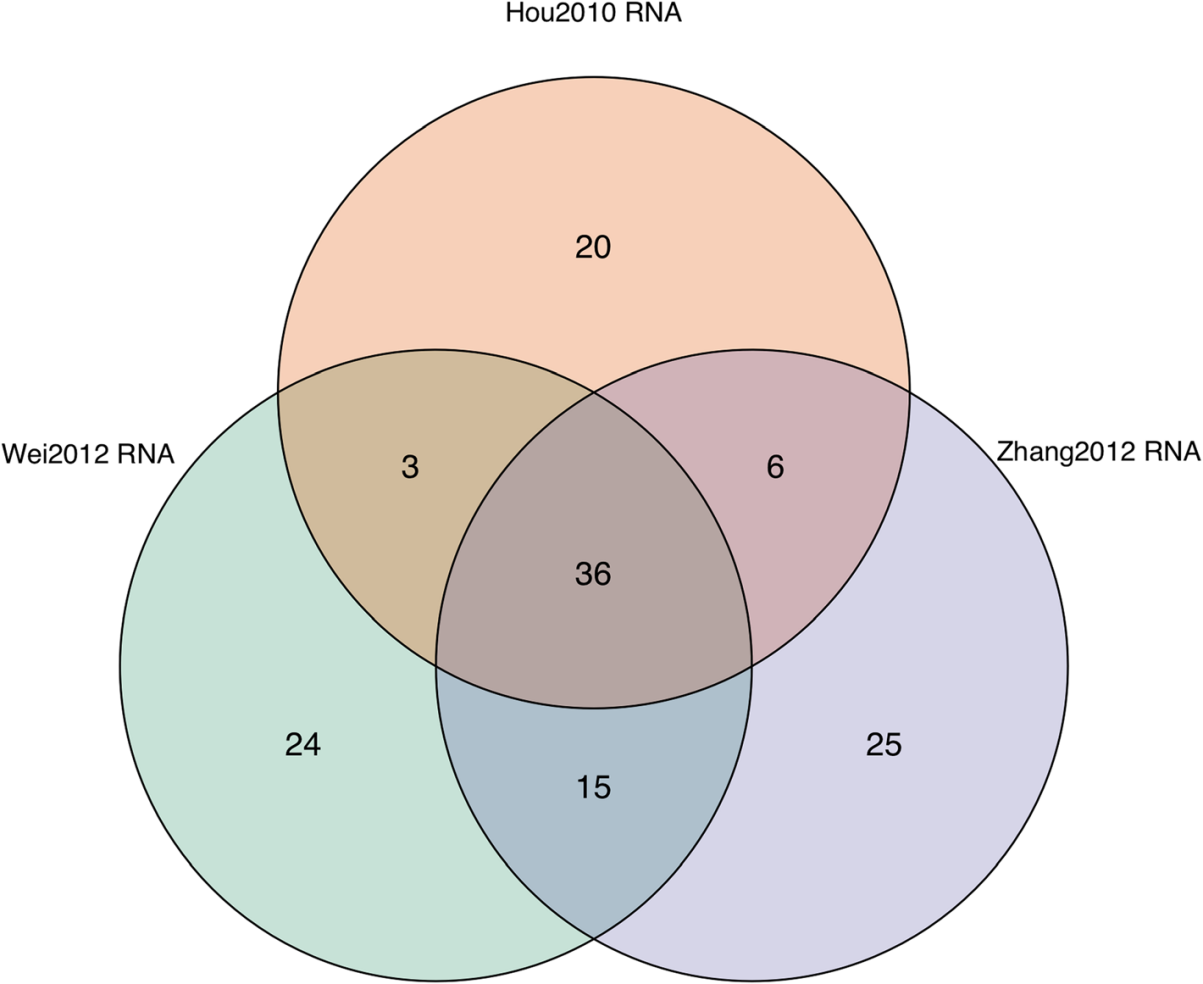
Venn diagram of the number of genes identified as differentially expressed in the Wei2012, Hou2010 and Zhang 2012 datasets

When the study design had been declared, a workflow was built to import and process transcriptomics data. The workflow imported data from the miodindata package using importProcessedData followed by processMicroarrayData to remove genes with an expression below 5 in all samples. The three datasets were integrated using integrateAssays and linear modeling with limma [19] was carried out with performOmicsModeling. This module identifies differentially expressed genes in each individual dataset and by setting metaAnalysis to TRUE an additional step is performed to reveal concordant results between the datasets. The final results are stored as a Venn diagram accompanied by data frames, which can be exported to disk (data frames are exported as Excel sheets). The Venn diagram is shown in Fig. 3 and the list of differentially expressed genes is provided in Additional file 2. The analysis script is available in Additional file 1.

### Vertical integration: exploratory data analysis on multi-omics data

The TCGA data used for vertical integration contained 338 breast cancer samples, for which survival status (alive or diseased) was available. To perform vertical integration, a case-control study design was declared and one assay table for each omics modality was added, specifying the data files to import. A workflow was built to import data with importProcessedData, followed processSequencingData to perform RNA-seq count filtering and variance stabilization with DESeq2 [46]. Methylation data was processed with processMicroarrayData to remove probes flagged as problematic by DMRcate [47] and non-CpG probes. The multi-omics data was integrated with integrateAssays and integrative analysis carried out by fitting a MOFA model to the data with performFactorAnalysis. The analysis script is available in Additional file 3.

**Fig. 4:**
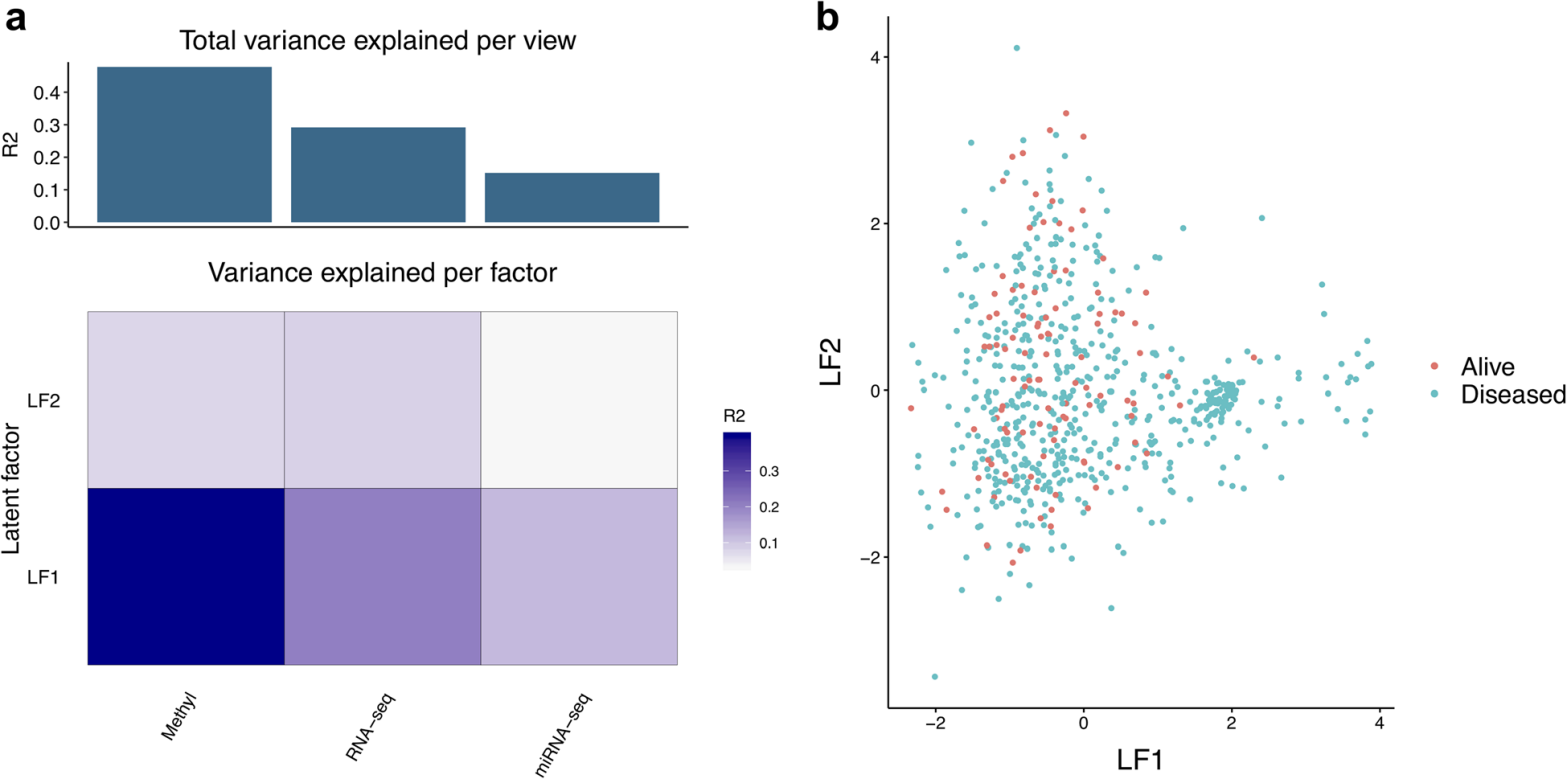
Assessment of the fitted MOFA model. **a** shows the total amount of variance explained by the model in each omics modality (view) and variance explained per factor. **b** shows a sample ordination plot based on latent factor (LF) 1 and 2

**Fig. 5:**
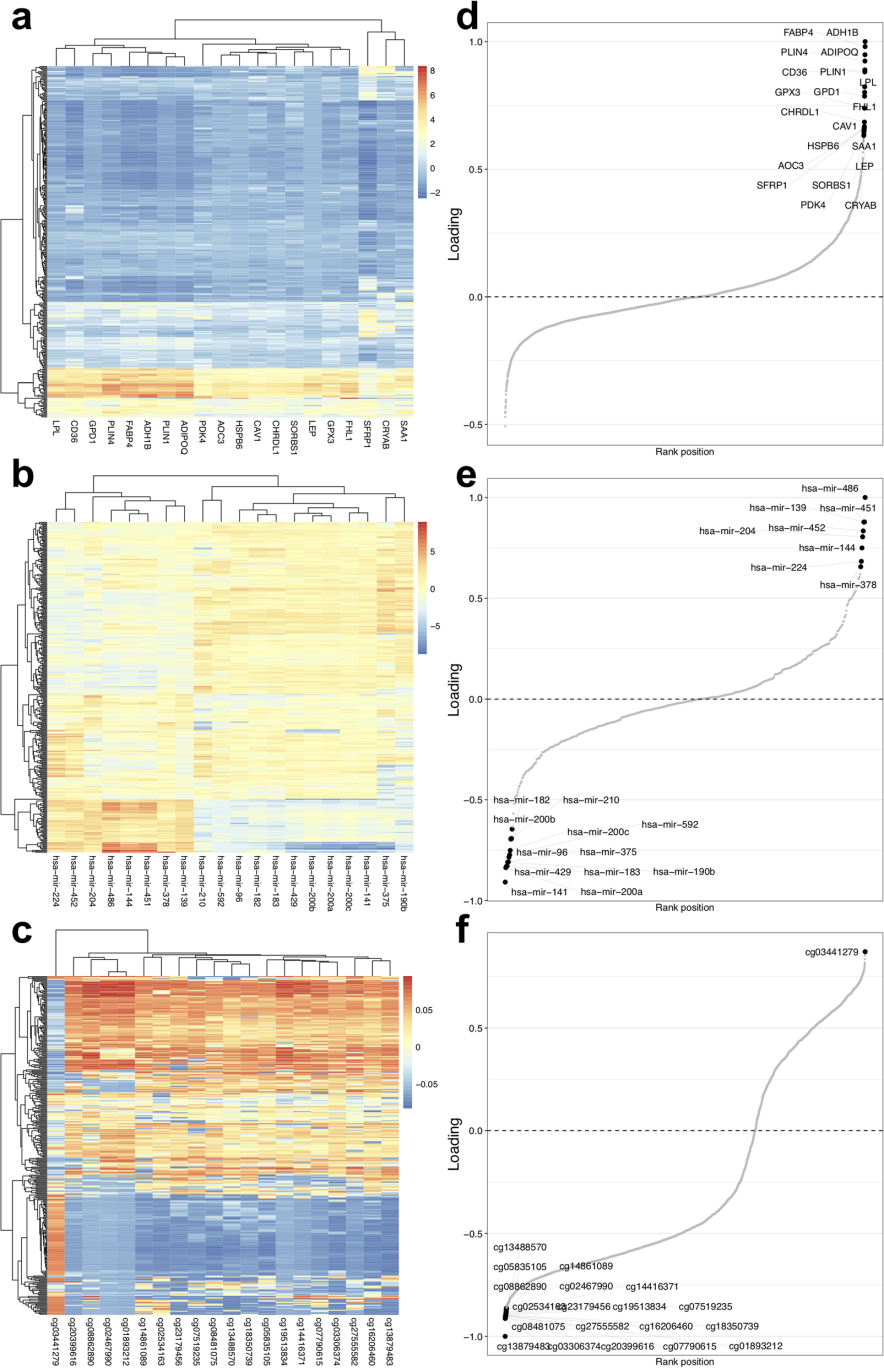
Interpretation of MOFA factor 1. **a** through **c** show sample heatmaps with the top features in the factor for RNA-seq gene, miRNA and 450 k methylation data, respectively. **d** through **f** reveal the loadings of the top features corresponding to the heatmaps in **a** through **c**

The results from performFactorAnalysis include the fitted model object and plots to assess the model in terms of variance explained, sample clustering (Fig. 4) and the top features in the first factor (Fig. 5). Plots for other factors can be rendered and further downstream analysis carried out with the model object. Jupyter notebooks for reproducing the horizontal and vertical analyses are provided in GitLab (https://gitlab.com/algoromics/miodin-notebooks), with the option to run on Binder [33].

## Discussion

Multi-omics experiments are indispensable for studying biological systems across molecular layers. In order to capitalize on the availability of high-throughput data and perform integrative analyses, analysts need to develop complex pipelines that can incorporate methods for import, processing and integration of different data types. Thanks to the rapid development of new bioinformatics tools, a large number of methods and software packages exist for various analysis problems. The difficulty lies in constructing a workflow that ensures transparency, scalability, reproducibility and tracking of data provenance during the analysis. A transparent workflow should reveal what main computational steps are performed and with what parameters. This helps the analyst to understand what happens to the data and how to interpret the results. Scalability implies that the workflow should cope with very large datasets, e.g. when analyzing genome-wide DNA variants or methylation patterns. To address this, it should be possible to deploy the workflow on high-performance computer resources. Reproducibility implies that the workflow will generate the same results, given the same input data, when the analysis is rerun. Many results in the biomedical literature cannot be reproduced [48] and a major difficulty behind this is differences in the local execution environment, e.g. software dependencies and package versions [49]. Tracking of provenance is related to reproducibility and requires capture of information on what software packages have been used, versions, parameters and data produced during workflow execution [27, 50].

With the miodin package, users can build streamlined analysis workflows that address the aforementioned concerns. Transparency is achieved with a clean syntax where the user only needs to specify the main steps as workflow modules, along with any necessary parameters. This makes analysis script much shorter compared to when the same analysis is implemented from scratch, since the necessary programming logic is wrapped in the modules. The steps of the workflow become visible to the analyst without having to interpret every command in a large chunk of code. Scalability is supported by translating a miodin workflow into a Nextflow [29] script, which can be deployed as a job on high-performance computer clusters. This is enabled by simply setting deployAsJob = TRUE when calling execute on the workflow, provided that Nextflow is installed (see the user manual for details). Job progress can be monitored in R with jobStatus and the workflow configured to notify the user by email when the job is finished.

To ensure workflow reproducibility, a Docker image called miodin-notebook was configured and uploaded to DockerHub. The image can be downloaded and used to run miodin locally or when deploying workflows as jobs. Nextflow supports Docker, so the user only needs to specify the name of the image with jobContainer when executing the workflow. By running the analysis within a pre-configured container, the software environment remains constant across different systems and ensures that results can be reproduced exactly. This makes it simple to externally verify the results without spending time on configuring dependencies. Tracking data provenance also becomes easy with miodin, since the workflow modules that import, process and analyze data will automatically add steps to the dataset’s processing protocol. These can be inspected in R or exported to Excel sheets by calling export on the dataset. This helps the analyst understand how processing steps have affected the data and to adjust parameters if necessary, prior to downstream analysis.

Several future developments are planned to enhance the functionality of the miodin package. Firstly, additional omics data types and platforms (e.g. raw sequencing and proteomics data, metabolomics, single cell, qPCR) will be supported. Secondly, several statistical and high-level analysis methods (e.g. clustering, classification, networks, annotation enrichment) will be implemented. Thirdly, workflow modules will be added for obtaining data from additional public repositories for omics, interaction and annotation data.

## Conclusions

This paper presented the miodin package, which provides an infrastructure for integration and analysis of multi-omics data. Key features include a high-level user API, an expressive vocabulary for declaring study designs, streamlined workflows and support for multiple omics data types and platforms. The package has been designed to promote transparent data analysis and supports scalability, reproducibility and tracking provenance during workflow execution. Jupyter notebooks are available online and can also be executed on Binder, which provides an accessible web-based interface for developing and testing workflows. To ensure the research community benefits from miodin, the software package with extensive documentation is made freely available on GitLab under the GPL-3 license.

### Availability and requirements

**Project name:** miodin

**Project home page:** https://gitlab.com/algoromics/miodin

**Operating system(s):** Windows, Linux, MacOS

**Programming language:** R

**Other requirements:** Python

**License:** GNU General Public License v3.0

**Any restrictions to use by non-academics:** No

## Supplementary information

**Additional file 1.** Horizontal integration analysis script. R script for performing horizontal integration as presented in the paper.

**Additional file 2.** Differentially expressed genes from meta-analysis. List of genes found differentially expressed in horizontal integration analysis.

**Additional file 3.** Vertical integration analysis script. R script for performing vertical integration as presented in the paper.

## Data Availability

Source code and user manual for the miodin package are available on GitLab (https://gitlab.com/algoromics/miodin). Additional file 1 contains the analysis script for horizontal integration. Additional file 2 contains the list of differentially expressed genes identified in horizontal integration analysis. Additional file 3 contains the analysis script for vertical integration. Processed datasets used for analysis are available as part of the miodindata companion package, also available on GitLab (https://gitlab.com/algoromics/miodindata). Source datasets for horizontal integration are available from ArrayExpress with accession numbers E-GEOD-27262, E-GEOD-19188 and E-GEOD-40791. Source datasets for vertical integration are available in the curatedTCGAData package from Bioconductor, 10.18129/B9.bioc.curatedTCGAData.

## Abbreviations

HCC: Hepatocellular carcinoma
LF: Latent factor
MOFA: Multi-omics factor analysis
TCGA: The Cancer Genome Atlas
